# Genome Sequences of Eight Bacterial Species Found in Coculture with the Haptophyte *Chrysochromulina tobin*

**DOI:** 10.1128/genomeA.01162-16

**Published:** 2016-11-03

**Authors:** Kathryn R. Fixen, Shawn R. Starkenburg, Blake T. Hovde, Shannon L. Johnson, Chloe R. Deodato, Hajnalka E. Daligault, Karen W. Davenport, Caroline S. Harwood, Rose Ann Cattolico

**Affiliations:** a Department of Microbiology, University of Washington, Seattle, Washington, USA; b Los Alamos National Laboratory, Los Alamos, New Mexico, USA; c Department of Biology, University of Washington, Seattle, Washington, USA

## Abstract

The microalgal division Haptophyta uses a range of nutritional sourcing, including mixotrophy. The genome of a member of this taxon, *Chrysochromulina tobin*, suggests that interactions with its bacterial cohort are critical for *C. tobin* physiology. Here, we report the genomes of eight bacterial species in coculture with *C. tobin*.

## GENOME ANNOUNCEMENT

Haptophytes encompass a large assemblage of microalgae that are abundant in both marine waters and freshwater environments, where they play an important role in global carbon sequestration (1, 2). The ecosystem dominance of these planktonic eukaryotes may be augmented by their mixotrophic ability to supplement photosynthesis with the acquisition of dissolved organic molecules, inorganic molecules, and particulate matter (including whole cells) from their surrounding medium (3).

Several haptophyte species have been observed to actively hunt bacteria using a specialized flagella-like appendage unique to haptophytes called the haptonema, suggesting that interaction of haptophytes with their eco-cohorts plays an important role in their physiology. This conclusion is further supported by recent sequencing of the genome of the haptophyte *Chrysochromulina tobin*. A notable observation is the putative dependence of *C. tobin* on its eco-cohorts for exogenous B_12_ acquisition, given that the alga only encodes a B_12_-dependent methionine synthase (4). To gain further insight into the metabolic interplay between *C. tobin* and its bacterial cohort, we carried out whole-genome sequencing of eight bacterial species of the nine identified bacteria present in coculture with this alga (Table 1).

**TABLE 1:** Genome features and GenBank accession numbers for eight bacterial species isolated in coculture with *Chrysochromulina tobin*

Organism	Accession no.	Genome size (bp)	Total no. of genes	No. of plasmids (plasmid accession no.)	Plasmid size (bp)	No. of plasmid-encoded genes	Fold coverage
*Acidovorax* sp. RAC01	CP016447	4,574,859	4,162	0	0		198
*Sinorhizobium* sp. RAC02	CP016450	6,626,583	6,327	1 (CP016451)			157
					258,555	566	
*Sphingobium* sp. RAC03	CP016456	4,369,746	4,207	3 (CP016455, CP016454, CP016457)			242
					63,550	74	
					49,737	52	
					131,734	141	
*Blastomonas* sp. RAC04	CP016460	4,403,499	4,178	4 (CP016461, CP016459, CP016458, CP016462)			298
					39,932	44	
					42,134	52	
					152,317	156	
					186,489	189	
*Bosea* sp. RAC05	CP016463	5.620,120	5,431	0	0		118
*Agrobacterium* sp. RAC06	CP016499	4,964,647	4,777	1 (CP016500)			329
					323,507	327	
*Hydrogenophaga* sp. RAC07	CP016449	4,674,684	4,415	0	0		307
*Methyloversatilis* sp. RAC08	CP016448	3,937,190	3,610	0	0		305

Bacteria were isolated from a laboratory-maintained *C. tobin* P3 culture, grown in proprietary RAC5 medium (4). The bacteria were isolated using RAC5 medium supplemented with 0.2% yeast extract, 0.5% casamino acids, and 0.1% glycerol. For whole-genome sequencing, genomic DNA from each isolate was extracted using the Qiagen Puregene yeast/bacteria kit B. A Pacific Biosciences (5) single-molecule long-read library was constructed and sequenced for each bacterial isolate according to the manufacturer’s instructions. All genomes were assembled using HGAP version 2.2.0 (6) and were checked for misassemblies by mapping the data back to the consensus sequences using BridgeMapper version 2.3.0 (https://github.com/PacificBiosciences/Bioinformatics-Training/wiki/Bridgemapper) and were corrected using Consed (7). Annotations of corrected assemblies were completed using an Ergatis workflow with minor manual curation (8). Bacterial taxonomic affinity was assessed by aligning PCR-recovered and genome-sequence-acquired 16S ribosomal DNA sequences using the SILVA Incremental Aligner (SINA) program (9). Six isolates are *Alphaproteobacteria* (*Sinorhizobium* sp. RAC02, *Sphingobium* sp. RAC03, *Blastomonas* sp. RAC04, *Bosea* sp. RAC05, *Agrobacterium* sp. RAC06, and *Methyloversatilis* sp. RAC08) and two isolates are *Betaproteobacteria* (*Acidovorax* sp. RAC01 and *Hydrogenophaga* sp. RAC07). Recovery of insufficient biomass precluded sequencing the genome of the final isolate, a *Bacteroidetes* sp. with >99% identity to *Leadbetterella* spp*.* Three of the eight bacterial isolates sequenced (*Sinorhizobium* sp. RAC02, *Bosea* sp. RAC05, and *Agrobacterium* sp. RAC08) encode the aerobic pathway for B_12_ biosynthesis. It is unclear if these bacterial isolates produce B_12_ in laboratory cultures of *C. tobin* since exogenous B_12_ is provided in the algal culture medium. Further studies are needed to determine if these isolates play a role in providing B_12_ to *C. tobin* under conditions where B_12_ is limited.

### Accession number(s).

The assembled and annotated genome sequences were deposited in GenBank under the accession numbers listed in Table 1.
